# *Leishmania braziliensis* exosomes activate human macrophages to produce proinflammatory mediators

**DOI:** 10.3389/fimmu.2023.1256425

**Published:** 2023-09-27

**Authors:** Fabio C. Peixoto, Dalila L. Zanette, Thiago M. Cardoso, Mauricio T. Nascimento, Rodrigo C. O. Sanches, Mateus Aoki, Phillip Scott, Sérgio C. Oliveira, Edgar M. Carvalho, Lucas P. Carvalho

**Affiliations:** ^1^ Laboratório de Pesquisas Clínicas (LAPEC), Instituto Gonçalo Moniz (IGM), Oswaldo Cruz Foundation (FIOCRUZ), Salvador, Bahia, Brazil; ^2^ Programa de Pós Graduação em Ciências da Saúde, Universidade Federal da Bahia, UFBA, Salvador, Bahia, Brazil; ^3^ Laboratory for Applied Science and Technology in Health, Instituto Carlos Chagas – Oswaldo Cruz Foundation (FIOCRUZ) Paraná (ICC), Curitiba, Paraná, Brazil; ^4^ Departamento de Bioquímica e Imunologia, Universidade Federal de Minas Gerais, Belo Horizonte, Minas Gerais, Brazil; ^5^ University of Pennsylvania, School of Veterinary Medicine, Philadelphia, PA, United States; ^6^ Departamento de Imunologia, Instituto de Ciencias Biomédicas, Universidade de São Paulo, São Paulo, Brazil; ^7^ Instituto Nacional de Ciências e Tecnologia-Doenças Tropicais, Salvador, Brazil

**Keywords:** Leishmania braziliensis, exosome, macrophage, immune response, innate immunity

## Abstract

Exosomes, organelles measuring 30-200nm, are secreted by various cell types. *Leishmania* exosomes consist of many proteins, including heat shock proteins, annexins, Glycoprotein 63, proteins exerting signaling activity and those containing mRNA and miRNA. Studies have demonstrated that *Leishmania donovani* exosomes downregulate IFN-γ and inhibit the expression of microbicidal molecules, such as TNF and nitric oxide, thus creating a microenvironment favoring parasite proliferation. Despite lacking immunological memory, data in the literature suggest that, following initial stimulation, mononuclear phagocytes may become “trained” to respond more effectively to subsequent stimuli. Here we characterized the effects of macrophage sensitization using *L. braziliensis* exosomes prior to infection by the same pathogen. Human macrophages were stimulated with *L. braziliensis* exosomes and then infected with *L. braziliensis*. Higher levels of IL-1β and IL-6 were detected in cultures sensitized prior to infection compared to unstimulated infected cells. Moreover, stimulation with *L. braziliensis* exosomes induced macrophage production of IL-1β, IL-6, IL-10 and TNF. Inhibition of exosome secretion by *L. braziliensis* prior to macrophage infection reduced cytokine production and produced lower infection rates than untreated infected cells. Exosome stimulation also induced the consumption/regulation of NLRP3 inflammasome components in macrophages, while the blockade of NLRP3 resulted in lower levels of IL-6 and IL-1β. Our results suggest that *L. braziliensis* exosomes stimulate macrophages, leading to an exacerbated inflammatory state that may be NLRP3-dependent.

## Introduction

Cutaneous leishmaniasis (CL), due to *L. braziliensis* infection, is characterized by the presence of few or rare parasites, a predominance of lymphocytes and mononuclear phagocytes in the inflammatory infiltrate (1). Host immunological factors are known to play an important role in the pathogenesis of this disease. Mononuclear cells from CL patients stimulated with soluble *Leishmania* antigen (SLA) produce exacerbated amounts of IFN- γ and TNF, as well as low levels of IL-10 in culture (2). While the production of IFN-γ is important for parasite killing, high levels of TNF have been associated with tissue damage and lesion development (2, 3). Studies on lesion tissue samples have confirmed the contribution of inflammation to ulcer development, as evidenced by the presence of Granzyme B produced by CD8+ and NK cells, in addition to metalloproteinases and inflammatory cytokines (4–11). Moreover, *Leishmania braziliensis*-infected mononuclear phagocytes have been shown to produce high levels of reactive oxygen species (ROS), molecules with leishmanicidal properties that have also been associated with tissue damage (12, 13).

An important role of IL-1β in CL pathogenesis has been documented in recent years, with the use of IL-1β inhibitors shown to improve the course of disease in murine models (8, 12). In humans, IL-1β concentrations were found to correlate with lesion size, and previous research by our group has documented elevated expression of the NLRP3 inflammasome in monocytes obtained from the peripheral blood of CL patients, in addition to high levels of IL-1β in cultured peripheral blood mononuclear cells (PBMC) stimulated with SLA (8, 14). Moreover, NLRP3^-/-^ BALB/c mice infected with *L. major* demonstrated a greater ability to control *Leishmania* infection when compared to wild-type mice (15).

*Leishmania*, and its soluble products, can trigger immune responses in innate immune cells prior to the onset of mononuclear cellular infection (13, 16, 17). For instance, *Leishmania* lipophosphoglycan (LPG) and DNA can induce the production of inflammatory mediators by mononuclear phagocytes through the activation of toll-like receptors (TLR) 2, 4 and 9 (18–20). Pathogen molecules are often secreted via extracellular vesicles (EV), also known as exosomes.

Exosomes, organelles ranging in size from 30-200nm, are secreted by various cell types. The invagination of regions of the endosomal membrane results in the formation of multivesicular bodies (MVB); the fusion of MVBs with the plasm membrane leads to exosome secretion (21, 22). These vesicles play a major role in molecular trafficking, e.g., proteins and nucleic acids, which once delivered may modulate host macrophage function (23–29). For example, glycoprotein (gp) 63-containing *Leishmania* exosomes was shown to induce TNF production by macrophages and exacerbate pathology in a CL mouse model (28, 30–32).

The present study aimed to assess the effects of *L. braziliensis* exosomes on human macrophagic responses, revealing that these vesicles induce both IL-1β and IL-6 production. Moreover, the pre-exposure of macrophages to *L. braziliensis* exosomes was found to prime these cells to enhance the production of pro-inflammatory mediators in response to *L. braziliensis* infection, potentially contributing to the pathogenesis of disease.

## Methods

### Parasite culture

An *L. braziliensis* isolate (MHOM/BR/LTCP11245) previously obtained from a CL patient’s skin lesion was initially cultivated in biphasic medium (NNN). After isolation, parasites were identified by multilocus enzyme electrophoresis and cryopreserved in liquid nitrogen. Following selection, parasites were expanded and cultivated in Schneider’s culture medium (SIGMA) supplemented with 20% inactive fetal bovine serum (FBS) (GIBCO), 1% L-glutamine and antibiotics.

### L. braziliensis exosome isolation and characterization

**Figure 1:**
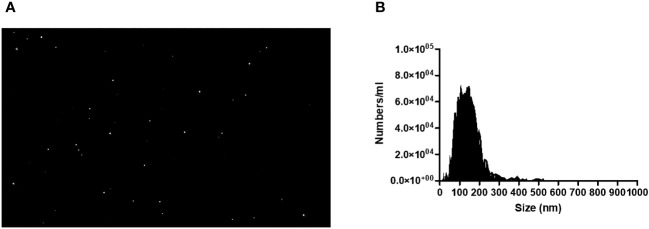
**(A)** Image of *L. Braziliensis* exosomes taken by a NS300 particle-sike tracker and Nanosight NTA 3.0 software using light scatter mode. **(B)** Quantification and Characterization of extracellular vesicles extracted from isolates of *L. braziliensis*.

*L. braziliensis* promastigotes were cultured at 37°C under 5% CO_2_ for 4 hours to optimize protein and vesicle secretion (31). Supernatants collected from *in vitro* cultures were centrifuged (2500rpm) and filtered (0.22 µm), after which exosomes were isolated through sequential ultracentrifugation (10 x 10^5^g). The EV particle-size distribution was determined by diffraction analysis using a NS300 particle-size tracker and Nanosight NTA 3.0 software using light scatter mode (Malvern Instruments Ltd., Technologies, Malvern, UK) (**Figure 1**) (33–35).

### Isolation and culturing of peripheral blood mononuclear cells

Peripheral blood mononuclear cells (PBMC) were obtained from heparinized venous blood from healthy subjects by Ficoll-paque density gradient centrifugation (GE Healthcare). Cells were washed twice in saline, and monocytes were isolated from PBMCs using magnetic beads in accordance with the manufacturer’s protocol (Dynabeads untouched human monocytes; Invitrogen Dynal AS, Oslo, Norway). This process was performed twice, after which cells were washed in phosphate-buffered saline (PBS), then adjusted to the desired concentration, and resuspended in RPMI 1640 (GIBCO BRL., Grand Island, NY USA) supplemented with 10% FBS (GIBCO BRL., Grand Island, NY USA) and antibiotics. Monocytes were then allowed to adhere to polystyrene culture plates and incubated for 6 days at 37°C under 5% CO_2_ until differentiation into macrophages, as previously described (36).

### Macrophage infection with L. braziliensis after stimulation with L. braziliensis soluble factors

**Figure 2:**
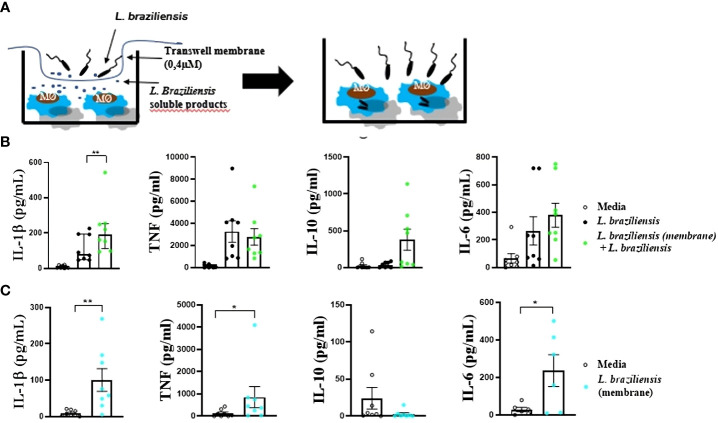
Macrophages from HS infected with *L. braziliensis* after sensitization with *L. braziliensis* through a transwell membrane (0,4µm). **(A)** Stimulation strategy. **(B)** Cytokine production by macrophages sensitized with soluble factors and infected with *L. braziliensis*. **(C)** Cytokine production by macrophages from HS sensitized with soluble factors from *L. braziliensis*. Levels of IL-1β, TNF, IL-10 and IL-6 were determined in culture supernatants by LUMINEX. Statistical analyses were performed using the Wilcoxon rank test *p<.05, **p<.01.

Monocyte-derived macrophages (3 x 10^5^) were stimulated, using a transwell membrane, with soluble factors from *L. braziliensis* for 24 hours at 37 °C, 5% CO_2_ in RPMI (**Figure 2A**). Following stimulation, cells were washed twice in saline and infected or not with *L. braziliensis* (MOI 5:1) for 2 hours. Next, the remaining promastigotes were washed out, and cells were reincubated for another 24 hours at 37 °C, 5% CO_2_ in RPMI. The slides were subsequently submitted to panoptic staining for posterior quantification of infected macrophages and amastigotes per 100 cells via optical microscopy.

### Exosome stimulation and macrophage infection with L. braziliensis

Monocyte-derived macrophages (3x10^5^) were stimulated with exosomes from *L. braziliensis* (300 vesicles/macrophage) for 24 hours at 37°C under 5% CO_2_ in RPMI. After stimulation, cells were washed twice in saline and infected or not with *L. braziliensis* (MOI 5:1) for 2 hours. The remaining promastigotes were washed out, then cells were incubated for another 24 hours (37 °C, 5% CO_2_) in RPMI.

### Parasite treatment with GW4869

For some experiments involving the inhibition of exosome/vesicle generation, 1x10^7^ *Leishmania*/mL were treated with GW4869 (20ng/ml), a vesicle generation inhibitor, (https://www.sigmaaldrich.com/BR/pt/product/sigma/d1692) for 30 minutes at room temperature, then washed three times to prevent contact between the inhibitor and human cells during stimulation/infection protocols. To assess GW4869 toxicity against *Leishmania* parasites, we evaluated *L. braziliensis* viability after treatment with GW4869 using propidium iodide as cell death marker, by flow cytometry.

### Macrophage treatment with glibenclamide

For the blockade of the NLRP3 inflammasome, monocyte-derived macrophages (3x10^5^) were first treated with the NLRP3 inhibitor glibenclamide (100µM) for 2 hours, then stimulated with L. braziliensis exosomes (300:1) for 24 hours. Cells were then washed twice and infected or not with L. braziliensis (MOI 5:1) for 2 hours, washed again to remove any remaining promastigotes, and finally incubated for 24 hours at 37 °C under 5% CO_2_in RPMI. Unstimulated macrophages and untreated stimulated cells were used as controls.

### Infection rate assessment

To evaluate infection rate, 3x10^5^ macrophages/well were plated on Nunc^®^ Labtek^®^ plates and stimulated with exosomes from *L. braziliensis* (300 vesicles/macrophage), then incubated for 24 hours at 37° C with 5% CO_2_. Cells were then washed twice and infected or not with *L. braziliensis* (MOI 5:1) for 2 hours, washed again to remove any remaining promastigotes, and finally incubated for 24 hours at 37 °C under 5% CO_2_ in RPMI. The slides were submitted to panoptic staining for the later quantification of infected macrophages and the number of amastigotes per 100 cells, performed via optical microscopy.

### Cytokine quantification

Following stimulation and/or infection protocols, the supernatants from cultures were collected for cytokine (IL-1β, TNF, IL-6 and IL-10) quantification via Luminex (Bio-Plex Pro Human Cytokine 27-plex Assay) or ELISA.

### Flow cytometry

Monocyte-derived macrophages from healthy subjects were stimulated with *L. braziliensis* exosomes for 24 hours and infected or not with *L. braziliensis* for another 24 hours, as described above. After the final incubation, cells were collected and placed in 5mL polystyrene FACS tubes (BD Biosciences Falcon™ 352052) for cell labeling with conjugated antibodies αCD14 (APC) and αHLA-DR (PerCP) to determine cell populations of interest, as well as αNLRP3 (PE). Events were acquired on a flow cytometer (BD LSRFortessa™ Cell analyzer) and data were analyzed using Flowjo^®^ software.

### Oxidative burst essay

To evaluate reactive oxygen species (ROS) production, macrophages were stimulated as described in “Exosome Stimulation and Macrophage infection with *L. braziliensis*” section, then treated with dihydrorhodamine-123 at 10ng/mL (Cayman Chemical Company) for 10 minutes. Cells were subsequently labeled with αHLA-DR and αCD14 to evaluate fluorescence intensity by flow cytometry, with data analysis performed via FlowJo^®^ software.

### RNA extraction, NF-κB and TLR2 gene expression

Cells stimulated with exosomes and infected or not with *L. braziliensis*, followed by incubation at 37°C under 5% CO_2_ for 2 hours, were harvested in TRIzol Reagent (Invitrogen). RNA extraction was performed using TRIzol RNA isolation, according to manufacturer’s instructions. RNA concentration and integrity were determined by spectrophotometric optical density measurements (260 and 280 nm). Gene expression was analyzed performed as previously described (37).

### Mouse macrophage cultures and infection

C57BL/6 mice, both wild-type and others genetically deficient for NLRP3^−/−^, were obtained as previously described (38). All animals were maintained at the UFMG Animal Facility and used for experimentation at 6–8 weeks of age. Bone marrow-derived macrophages (BMDM) were prepared and infected as previously described (39, 40). Briefly, bone marrow cells were isolated from the femurs and tibias of the animals, cultured in RPMI 1640 supplemented with 30% L929 cell-conditioned medium and 20% FBS for 7 days. BMDM (0.5×10^6^) were treated or not with lipopolysaccharide (LPS) for 6 hours (500 ng/ml) and stimulated or not with *L. braziliensis* exosomes (300:1), followed by infection with stationary phase *Leishmania braziliensis* (MOI 10:1) for 24 hours. After 48 hours, supernatants were harvested and IL-1β, TNF and IL-6 concentrations were detected by ELISA.

### Statistical analysis

Statistical analysis was performed using the Wilcoxon test for paired variables and Mann-Whitney rank test for unpaired measurements (*p<0.05, **p<0.01, ***p<0.001, ****p<0.0001). All experimental data were analyzed using Prism GraphPad^®^ 8.0.2, which was also used for graphical data representations.

## Results

### L. braziliensis soluble factors induce pro-inflammatory mediator production in human macrophages

Macrophages can be “trained” to enhance response to infection (41). For instance, macrophages exposed to *Saccharomyces cerevisiae* demonstrated an increased ability to produce TNF in response to TLR ligands, such as LPS (42). To investigate whether soluble factors from *L. braziliensis* would interfere in cytokine production, human macrophages were cultured with *L. braziliensis* separated by a membrane, which allowed only soluble factors/small molecules to cross the barrier. Macrophages exposed to *L. braziliensis* products through the membrane produced more IL-1β upon infection than those that were not previously exposed to parasite factors (**Figure 2B**). Moreover, exposure to *L. braziliensis* soluble factors was also shown to induce the production of inflammatory mediators TNF, IL-6 and IL-1β by uninfected macrophages (**Figure 2C**). This data provides evidence that exposure to *L. braziliensis* modulates immune response prior to the establishment of cellular infection.

### L. braziliensis exosomes induce pro-inflammatory mediator production by human macrophages

**Figure 3:**
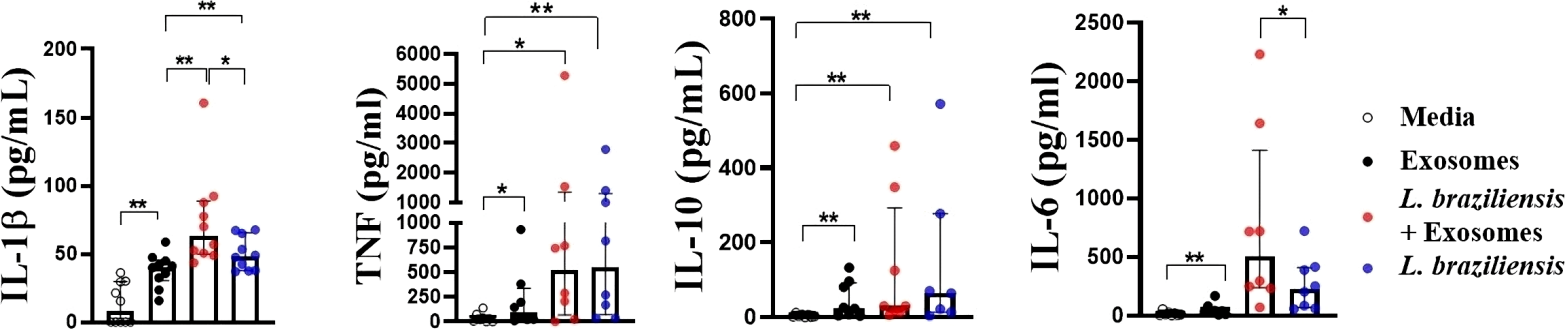
Cytokine production by macrophages from HS sensitized with *L. braziliensis* exosomes and infected with *L. braziliensis*. Macrophages from HS (n=8) were stimulated with exosomes isolated from *L. braziliensis* (300:1) for 24 hours and afterwards infected or not with *L. braziliensis* for another 24 hours. Levels of IL-1β, TNF, IL-10 and IL-6 were determined in culture supernatants by LUMNEX. Statistical analyses were performed using the Wilcoxon rank test *p<.05, **p<.01.

We first investigated whether *L. braziliensis* exosomes induced an inflammatory response in macrophages, and then assessed the effects of stimulating macrophages with these vesicles prior to infection with *L. braziliensis.* Compared to unstimulated cells, macrophages stimulation with EVs were found to induce IL-1β, TNF, IL-10 and IL-6 production (**Figure 3**). Pre-sensitization of macrophages with exosomes prior to infection was shown to increase IL-1β and IL-6 levels (**Figure 3**). Similarly, pre-sensitization with exosomes produced no influence on infection rate, nor the number of parasites internalized (data not shown).

### Blockade of L. braziliensis exosome generation inhibits Leishmania-induced cytokine production by human macrophages

**Figure 4:**
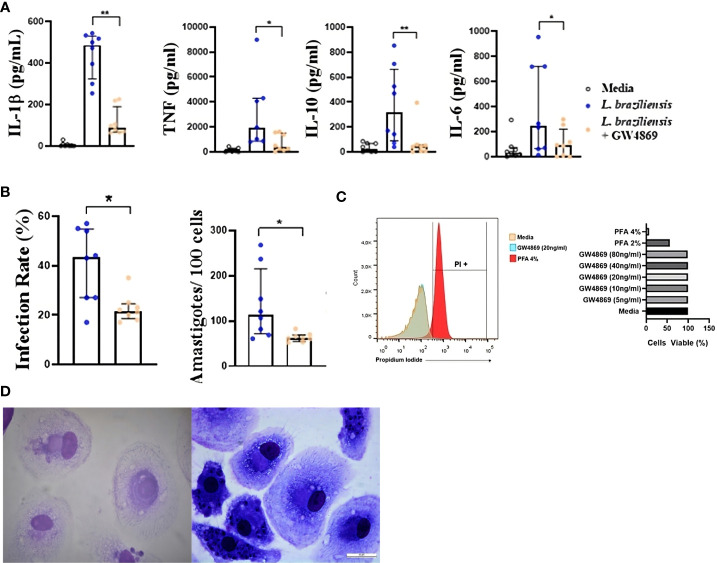
**(A)** Cytokine production and **(B)** Infection rate of macrophages from HS infected with *L. braziliensis* with exosome production inhibited. Axenic culture of *L. braziliensis* promastigotes was treated with GW4869 (20ng/ml), exosome production inhibitor, for 2 hours. Macrophages from HS (n=8) were infected with GW4869 treated *L. braziliensis* (5:1) for 24 hours. **(C)** *L. braziliensis* viability after GW4869 treatment was assessed using propidium iodide as cell death marker, by flow cytometry. **(D)** optical microscopy picture of panoptic stained macrophages infected with *L. braziliensis* treated (left) or not (right) with GW4869. Levels of IL-1β, TNF, IL-10 and IL-6 were determined in culture supernatants by LUMNEX. The percentage of infected cells as well as the number of intracellular parasites were determined by microscopic evaluation after panoptic staining. Statistical analyses were performed using the Wilcoxon rank test *p<.05, **p<.01.

To further ascertain the role of exosomes in inducing inflammatory cytokine production, we infected human macrophages with *L. braziliensis* previously treated with GW4869, and then compared cytokine expression with other untreated *Leishmania-*infected cells. As in the experiments above, our data shows that cells infected with exosome-free *L. braziliensis* produced significantly less IL-1β, TNF, IL-10 and IL-6 upon infection (**Figure 4A**). Although no differences were observed in parasite internalization by macrophages pre-sensitized with exosomes compared to unstimulated cells (data not shown), the cells infected with exosome-free *L. braziliensis* presented less infectivity, as evidenced by fewer numbers of infected macrophages and lower numbers of amastigotes within the cells (**Figure 4B**). As treatment with GW4869 could have interfered with *L. braziliensis* survival, we performed a dose-response curve with different GW4869 concentrations. Our experiments show no toxicity of GW4869 over *L. braziliensis* (**Figure 4C**).

### L. braziliensis exosomes induce NLRP3 consumption/regulation

**Figure 5:**
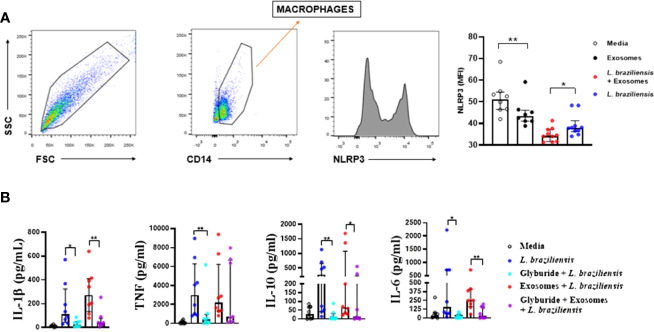
**(A)** Exosome stimulation enhance NLRP3 consumption by macrophages. Macrophages from healthy subjects (n=8) were sensitized with exosomes (300:1) for 24 hours and infected with *L. braziliensis* promastigotes for another 24 hours at a ratio of 5:1. Cells were stained with anti-CD14 and anti-NLRP3. Data were collected using flow cytometry and analyzed with FLOWJO^®^ software. Representative gating strategy on CD14^+^ expression in macrophages from one healthy subject. NLRP3 MFI was taken from CD14^+^ population. The data represent the mean of fluorescence intensity (MFI) of NLRP3 expression by macrophages in the different stimulated groups. **(B)** Glyburide downmodulates exosome-induced cytokine production in macrophages from healthy subjects. Macrophages from 8 individuals were treated with glyburide (100µM) for 2 hours. Afterwards cells were stimulated for 24 hours with *L. braziliensis* exosomes and infected with the parasite for another 24 hours. Levels of IL-1β, TNF, IL-10, IL-10 and IL-6 were measured in culture supernatant by ELISA. Statistical analyses were performed using the Mann-Whitney test for unpaired groups and Wilcoxon rank test for paired measurements *p<.05 **p<.01.

Most of the data presented in this study demonstrate the important association between *L. braziliensis* exosomes and IL-1β production. Consequently, the participation of NLRP3 in IL-1β production was investigated by evaluating both the expression of NLRP3 protein by cultured macrophages (**Figure 5A**) and the production of cytokines by cells stimulated with exosomes after the blockade of NLRP3 through glibenclamide treatment (**Figure 5B**). Our results indicate lower NLRP3 protein expression in cells stimulated with exosomes for 24 hours compared to basal level cells, while exosome stimulation in macrophages prior to infection induced more consumption, and probably further expression regulation, of this inflammasome than unsensitized cells (**Figure 5A**), which corroborates our previous results. Moreover, glibenclamide-treated cells were found to secrete less IL-1β, TNF, IL-6 and IL-10 than untreated cells, regardless of *L. braziliensis* infection or exosome stimulation. (**Figure 5B**), which suggests that cytokine expression induced by exosome stimulation is dependent on NLRP3.

### BMDM from NLRP3^-/-^ C57BL/6 mice exhibit inhibited IL-1ß production following. braziliensis infection

The secretion of IL-1β may be dependent on inflammasome activation (8, 14, 39). NLRP3, the main inflammasome receptor responsible for inducing IL-1β production, forms a complex with ASC and Caspase-1 for the processing and secretion of IL-1β and IL-18 (43, 44). Since our results showed that *L. braziliensis* exosome stimulation followed by infection induced high levels of IL-1β secretion by human macrophages, we decided to investigate whether NLRP3 activation is required to drive IL-1β production upon *L. braziliensis* infection in a murine model.

**Figure 6:**
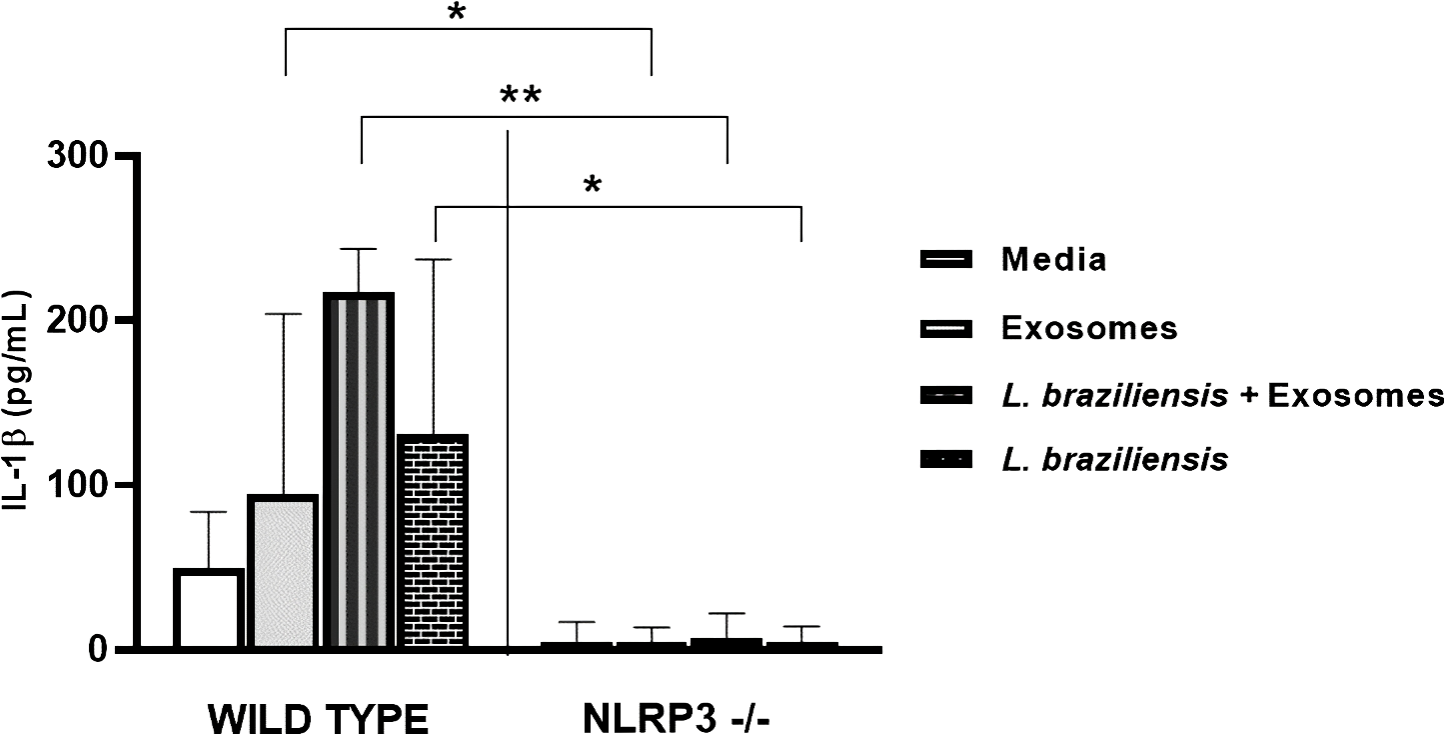
NLRP is key for *L. braziliensis* induced IL-1β production in mouse macrophages. BMDMs from wild-type C57BL/6 mices and deficient for NLRP3 were prepared, pulsed with LPS (500 ng/ml), stimulated with exosomes (300:1) and infected with *L. braziliensis* (MOI 10:1) or not. After 48 hours of culture ELISA for IL-1β was performed on supernatants. Statistical analyses were performed using the Mann-Whitney test and the Wilcoxon rank test. *p<0.05, **p<0.01.

Bone marrow-derived macrophages (BMDM) from C57BL/6 WT mice and mice deficient for NLRP3 were infected with *L. braziliensis* after previous stimulation with *L. braziliensis* exosomes. Levels of IL-1β, IL-6 and TNF were quantified by ELISA in cell supernatants. IL-1β production in mice was found to be dependent on the NLRP3 inflammasome, as IL-1β production was completely abrogated in cells from NLRP3^-/-^ mice, regardless of stimulation. However, NLRP3 did not appear to be involved in the production of the other cytokines evaluated, as levels did not differ between WT and NLRP3^-/-^ mouse cells (**Figure 6**).

### L. braziliensis exosomes induce ROS production by macrophages

**Figure 7:**
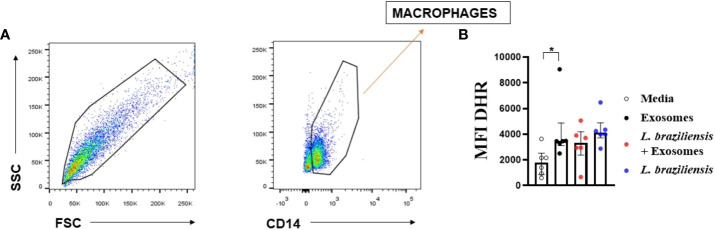
Macrophagic stimulation with *L. braziliensis* exosomes enhance reactive oxygen species production. Macrophages from healthy subjects were evaluated after being stimulated with exosomes for 24 hours and infected with *L. braziliensis* for 24 hours (n=6) or not. The cells were treated with DHR (10ng/mL – 10 min) and stained with anti-CD14 and anti-HLA-DR. Data were collected using flow cytometry and analyzed by FLOWJO^®^ software. **(A)** Representative gating strategy on CD14^+^ and HLA-DR^+^ expression in macrophages. DHR MFI were taken from CD14^+^ HLA-DR^+^ population. **(B)** The data represent the mean of fluorescence intensity (MFI) of oxidative burst production by macrophages stimulated with exosomes and infected with *L. braziliensis*. Statistical analyses were performed using the Wilcoxon rank test for paired measurements *p<.05.

Our group previously showed that reactive oxygen species (ROS) constitute a major endogenous factor in *Leishmania* killing (13, 45). Furthermore, TNF production is directly associated with ROS production through the activation of the NF-κB signaling pathway. Therefore, to investigate the role of *L. braziliensis* exosomes on ROS production, ROS expression (**Figure 7**) was evaluated in macrophages stimulated with exosomes further infected with *L. braziliensis*. Our data show that while *L. braziliensis* exosomes induced ROS production by uninfected macrophages, pre-sensitization did not interfere in ROS production following *in vitro* macrophage infection (**Figure 7**).

### Macrophages stimulated with L. braziliensis exosomes express high levels of NF-κB and toll-like receptor 2

**Figure 8:**
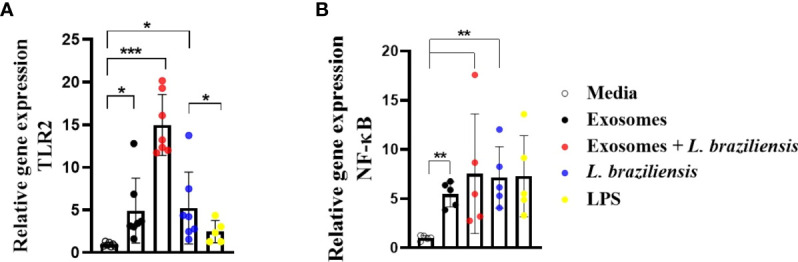
Macrophages stimulated with *L. braziliensis* exosomes express high levels of NF-κB and Toll-Like Receptor 2. **(A)** TLR2 and **(B)** NF-κ B gene expression, represented as 2^−ΔΔCT^, following RT-PCR of RNA from macrophages-derived monocytes of HS (N=7) stimulated with exosomes for 24 hours and infected with *L. braziliensis* for 24 hours or not. Statistical analyses were performed using the Mann-Whitney test and the Wilcoxon rank test. *p<0.05, **p<0.01. ***p<0.001.

Herein *L. braziliensis* exosomes were found to induce TNF and ROS production by human macrophages. It has been shown that NF-kB activation induces TNF production through the activation of TLRs 2-4 upon *L. braziliensis* infection (18). To determine the role of *L. braziliensis* exosomes in TLR2 and NFkB expression, human macrophages were stimulated with EVs for 2 hours and infected or not with *L. braziliensis* for another 2 hours. Uninfected macrophages stimulated with exosomes were found to induce higher expression of NF-κB and TLR2 (**Figure 8**), yet infected cells previously stimulated with EVs demonstrated increased TLR2 expression, yet no effect on NF-κB expression (**Figure 8**). No differences were observed among the groups with regard to TLR4 expression. These data corroborate with our findings on exosome-induced TNF and ROS production, as these molecules are formed as a result of the NF-κB pathway.

## Discussion

The initial events occurring upon *Leishmania* infection orchestrate immune response, constituting determinants in parasite proliferation and disease development. Among the factors contributing to disease expression, vector molecules, including salivary gland proteins, parasite-derived molecules and host immune response are of great importance (46, 47). On the parasite side, attention has been paid to the role of exosomes due to the close relationship between molecules present in the exosomes and their ability to modulate host immune response (28, 31, 32). The present study documented that *L. braziliensis* exosomes indeed modulate immune response by priming macrophages to produce more inflammatory molecules.

It has recently been suggested that macrophages could become “trained” to enhance infection response (41). For instance, macrophages exposed to *Saccharomyces cerevisiae* demonstrated an increased ability to produce TNF in response to TLR ligands, such as LPS. Moreover, monocytes “trained” with *S. cerevisiae* demonstrated heightened microbial activity (42). In the same vein, our results additionally indicate that *L. braziliensis* exosomes effectively “train” macrophages to produce higher cytokine concentrations in response to posterior *L. braziliensis* infection, suggesting that epigenetics in macrophages may play a role in subsequent exposure to *L. braziliensis* components.

Clearly, *Leishmania* vesicle contents vary across species, and thereby exert variable effects on host immune response. For instance, studies have shown that exosomes from *L. donovani* modulate monocyte response to IFN-γ and inhibit TNF while inducing IL-10 production. In this manner, mice stimulated with *L. donovani* exosomes prior to infection with the same species were found to exhibit higher macrophage infection rates within the spleen (26, 27). Also, stimulation with *L. major* exosomes prior to *L. major* parasite challenge produced a shift towards a Th2-type response in mice, as evidenced by a high frequency of CD4+ T cells producing IL-4, which led to disease exacerbation (27). Moreover, it was demonstrated that *L. major* exosomes contribute to CL pathology through the induction of an overproduction of inflammatory cytokines IL-23 and IL-17 in the lymph nodes of BALB/c mice (29).

The pathogenesis of *Leishmania* infection varies widely depending on the *Leishmania* species involved, and its specific effect on host immune response. In all cases, parasite control is associated with the expansion of CD4+ Th1 cells producing IFN-γ, which promotes the killing of parasites within infected cells (48). In some cases, the parasite evades the immune system silently, without inducing an immunological response, as is the case in infection by *L. donovani*. In *L. braziliensis* infection, however, an exaggerated Th1 response is observed, together with high levels of proinflammatory cytokines and a predominance of lymphocytes and mononuclear phagocytes at the lesion site. Herein we demonstrate that stimulation with *L. braziliensis* exosomes induces high levels of proinflammatory cytokines (TNF and IL-1β) as well as ROS, molecules known to be involved in CL due to *L. braziliensis* pathogenesis. In our experiments where we treated *L. braziliensis* with GW4869, a vesicle secretion inhibitor, we observed a decrease in cytokines production in infected macrophages. However, since GW4869 also decreased *L. braziliensis* internalization, further studies need to be performed to understand the mechanism by which *L. braziliensis* exosomes interfere in *Leishmania* uptake.

Some *Leishmania* species are known to inhibit several macrophage functions, such as macrophage activation, cytokine release and antigen presentation. For instance, down-regulation of class II MHC expression and the inability to produce IL-12 has been observed in several studies (49–54). Also, TLR-induced up-regulation of co-stimulatory molecules, as well as TNF-alpha and IL-12 production, was notably impaired in *L. major, L. chagasi, L. donovani* and *L. mexicana*-infected macrophages, while in the case of *L. mexicana*, disruption of NF-κB signaling was observed (55, 56). In contrast, our results show that exosome sensitization increased the expression of NF-κB and TLR2, corroborating previously published data indicating that *L. braziliensis* infection promotes an inflammatory environment.

Inflammation is often associated with macrophage activation and intracellular parasite killing. Here we found that in spite of *L. braziliensis* exosomes enhancing IL-1b production by human macrophages, these vesicles have no effect on Leishmania parasite killing. These results are in accordance with our previous data showing no association between IL-1b production and L. brazileisns killing (8). Also, in our current results we found that exosomes do not increase the ability of macrophages to produce ROS in response to L. braziliensis infection, corroborating that these vesicles have no effect on L. braziliensis killing by macrophages. In the present study we found that the IL-1b production driven by *L. braziliensis* vesicles is NLPR3-dependent, corroborating our previous data involving *L. braziliensis*-infected macrophages (8). These findings favor the potential role of *L. braziliensis* EVs in the immunopathology observed in CL arising from *L. braziliensis*, in which ulcer development has been associated with an exaggerated inflammatory response that leads to tissue damage. Previous results have shown that IL-1β concentrations in CL due to *L. braziliensis* correlate positively with lesion size and healing time (8, 12). It has been documented that *L. braziliensis* infection activates the NLRP3 inflammasome, thereby inducing ROS (8, 39). Therefore, the blockade of NLRP3 may constitute a sound therapeutic approach.

Considering the physiology and dynamics of vesicle secretion, studies involving *L. mexicana* demonstrated a substantial increase in vesicle secretion at a temperature of 37˚C (31). Herein we observed a similar phenomenon in *L. braziliensis*. The up-regulation in vesicle release induced at infection-like temperatures suggests that parasites release vesicles into the extracellular environment prior to invading host cells. Similarly, it has been suggested that *Leishmania* exosomes interact with the host cell prior to the parasite itself, as evidenced by the presence of *Leishmania* molecules within uninfected macrophages (57). Moreover, as previous studies have shown that *Leishmania* secretes exosomes into the midgut of the sandfly vector, it has been hypothesized that these vesicles then become inoculated into the host alongside the parasite during sandfly blood-feeding, possibly enhancing the vesicle-induced effects of the parasite on immune response (29, 32). Although both *L. major* and *L. braziliensis* cause CL, the magnitude of inflammation caused by both parasite species is quite different, as infection with *L. braziliensis* induces higher inflammatory response in humans than *L. major*. Thus, further experiments need to be performed to determine differences in the contents of exosomes between both species.

In contrast to previously published data indicating the downregulatory effects of *L. donovoni* and *L. major* exosomes, *L. braziliensis* exosomes do not appear to contribute to a microenvironment favorable for parasite growth, but instead participate in an exacerbated pathologic inflammatory response, which may potentially exacerbate lesion development.

## Data availability statement

The original contributions presented in the study are included in the article/supplementary materials. Further inquiries can be directed to the corresponding authors.

## Ethics statement

The studies involving humans were approved by Institution Review Board of the Federal University of Bahia Medical School and the National Commission of Ethics in Research (CONEP) under the number 3.252.513. The studies were conducted in accordance with the local legislation and institutional requirements. The human samples used in this study were acquired from Peripheral blood was collected from healthy volunteers. Written informed consent for participation was not required from the participants or the participants’ legal guardians/next of kin in accordance with the national legislation and institutional requirements. The animal study was approved by Institutional Animal Care and Use Committee of the Federal University of Minas Gerais (CEUA no. 165/2019). The study was conducted in accordance with the local legislation and institutional requirements.

## Author contributions

FP: Conceptualization, Data curation, Formal Analysis, Investigation, Methodology, Writing – original draft, Writing – review & editing. DZ: Investigation, Methodology, Formal Analysis, Writing – review & editing. TC: Formal Analysis, Methodology, Writing – review & editing. MN: Investigation, Methodology, Writing – review & editing. RS: Methodology, Resources, Writing – review & editing. MA: Investigation, Writing – review & editing. PS: Funding acquisition, Resources, Visualization, Writing – review & editing. SO: Resources, Writing – review & editing. EC: Formal Analysis, Funding acquisition, Project administration, Resources, Supervision, Visualization, Writing – review & editing. LC: Conceptualization, Data curation, Formal Analysis, Funding acquisition, Investigation, Project administration, Resources, Supervision, Visualization, Writing – original draft, Writing – review & editing.
